# Differential Expression of Genes Regulating Store-operated Calcium Entry in Conjunction With Mitochondrial Dynamics as Potential Biomarkers for Cancer: A Single-Cell RNA Analysis

**DOI:** 10.3389/fgene.2022.866473

**Published:** 2022-05-31

**Authors:** Mangala Hegde, Uzini Devi Daimary, Sandra Jose, Anjana Sajeev, Arunachalam Chinnathambi, Sulaiman Ali Alharbi, Mehdi Shakibaei, Ajaikumar B. Kunnumakkara

**Affiliations:** ^1^ Cancer Biology Laboratory, Department of Biosciences and Bioengineering, Indian Institute of Technology-Guwahati, Guwahati, India; ^2^ DBT-AIST International Center for Translational and Environmental Research, Indian Institute of Technology-Guwahati, Guwahati, India; ^3^ Department of Botany and Microbiology, College of Science, King Saud University, Riyadh, Saudi Arabia; ^4^ Musculoskeletal Research Group and Tumor Biology, Faculty of Medicine, Institute of Anatomy, Ludwig-Maximilian-University Munich, Munich, Germany

**Keywords:** head and neck cancer, store-operated calcium channels, mitochondrial dynamics, TCGA database, CPTAC database, gene expression omnibus

## Abstract

Regulation of intracellular concentration of calcium levels is crucial for cell signaling, homeostasis, and in the pathology of diseases including cancer. Agonist-induced entry of calcium ions into the non-excitable cells is mediated by store-operated calcium channels (SOCs). This pathway is activated by the release of calcium ions from the endoplasmic reticulum and further regulated by the calcium uptake through mitochondria leading to calcium-dependent inactivation of calcium-release activated calcium channels (CARC). SOCs including stromal interaction molecules (STIM) and ORAI proteins have been implicated in tumor growth, progression, and metastasis. In the present study, we analyzed the mRNA and protein expression of genes mediating SOCs—STIM1, STIM2, ORAI1, ORAI2, ORAI3, TRPC1, TRPC3, TRPC4, TRPC5, TRPC6, TRPC7, TRPV1, TRPV2, TRPM1, and TRPM7 in head and neck squamous cell cancer (HNSC) patients using TCGA and CPTAC analysis. Further, our *in silico* analysis showed a significant correlation between the expression of SOCs and genes involved in the mitochondrial dynamics (MDGs) both at mRNA and protein levels. Protein-protein docking results showed lower binding energy for SOCs with MDGs. Subsequently, we validated these results using gene expression and single-cell RNA sequencing datasets retrieved from Gene Expression Omnibus (GEO). Single-cell gene expression analysis of HNSC tumor tissues revealed that SOCs expression is remarkably associated with the MDGs expression in both cancer and fibroblast cells.

## Introduction

Calcium (Ca^2+^) is a ubiquitous intracellular second messenger that controls a wide range of physiological and pathological processes (Berridge et al., 2003). In metazoans including humans, the store-operated calcium channel entry (SOCE) is the predominant calcium entry mechanism in non-excitable cells (Parekh and Putney, 2005). The concept of SOCE was first described by James Putney in 1986; however, the molecular signatures and functional validation of SOCE were identified more recently (Putney, 1986). SOCs are activated when Ca^2+^ is released from the endoplasmic reticulum, which is necessary for cells to replenish Ca^2+^ after signaling processes (Parekh and Putney, 2005). SOCE is implicated in a wide range of biological processes such as transcriptional regulation of gene expression, exocytosis, cellular metabolism, and cell motility (Lewis, 2007; Parekh and Putney, 2005). Among the SOCs, STIM1, and ORAI1 have been shown to be widely expressed. Additionally, closely associated channel subfamilies like transient receptor potential channels (TRPC), TRPV (vanilloid), and TRPM (melastatin) have also been shown to be involved in SOCE mediated Ca^2+^ influx (Authi, 2007; Ma et al., 2011; Bastián-Eugenio et al., 2019). A plethora of studies showing the involvement of Ca^2+^ in various stages of cancer development and progression led Yang et al. (2009) to investigate the role of STIM1 and ORAI1 in breast cancer cell migration, invasion, and metastasis (Yang et al., 2009). In addition, transcriptomic analysis of glioblastoma tumor tissues showed overexpression of STIM1, ORAI1, and TRPC1 (Scrideli et al., 2008; Alptekin et al., 2015). Moreover, Zhang et al. (2013) demonstrated that SOCE mediated influx of Ca^2+^ regulated the migration and metastasis of nasopharyngeal carcinoma both *in vitro* and in zebrafish models (Zhang et al., 2013). Further, Jiang et al. (2007) revealed that TRPM7 is necessary for the proliferation and growth of FaDu and SCC25 cells *in vitro* by siRNA-mediated knockdown of TRPM7 (Jiang et al., 2007). SOCs have also been proposed as potential therapeutic targets for various inflammatory disorders and cancer (Feske, 2019; Khan et al., 2020; Chang et al., 2021). Recent studies have shown that non-steroidal anti-inflammatory drugs (NSAIDs) including sulindac, salicylate, flurbiprofen, and indomethacin inhibited SOCEs in colon cancer cells (Hernández-Morales et al., 2017; Villalobos et al., 2019). Furthermore, Gualadani et al. (2019) demonstrated the requisite of SOCE for the anti-cancer effect of cisplatin in non-small cell lung carcinoma (Gualdani et al., 2019). Therefore, these studies suggest that SOCs can be used as cancer diagnostic biomarkers and therapeutic targets.

According to the latest GLOBOCAN report on cancer burden worldwide, the prevalence of head and neck cancer is steadily increasing (Sung et al., 2021). Apart from the well-known risk factors such as tobacco, alcohol, and human papillomavirus (HPV), vitamin D insufficiency and defects in calcium signaling have recently been found to play a significant role in the initiation and progression of head and neck cancer (Orell-kotikangas et al., 2012; Singh et al., 2020).

Recently, studies have shown that ORAI, ORAI2, and STIM1 were significantly elevated in tissues from oral squamous cancer patients compared to normal samples. These studies also reported the significant reduction in proliferation, migration, and invasion upon siRNA-mediated knockdown of ORAI1, ORAI2, and STIM1 in oral cancer cell lines *in vitro* (Singh et al., 2020; Wang et al., 2022). In the present study, we analyzed the gene and protein expression of SOCs—STIM1, STIM2, ORAI1, ORAI2, ORAI3, TRPC1, TRPC3, TRPC4, TRPC5, TRPC6, TRPC7, TRPV1, TRPV2, TRPM1, and TRPM7 in HNSC using The Cancer Genome Atlas (TCGA) and Clinical Proteomic Tumor Analysis Consortium (CPTAC) analysis. In addition, mitochondrial regulation of calcium ions has shown to play an important role in SOCs mediated calcium entry, and tumor cell mitochondrial dysfunction is proposed to be responsible for SOCs upregulation (Villalobos et al., 2018). Hence, we further analyzed the genes involved in mitochondrial dynamics (MDGs) and found a significant correlation between the expression of SOCs and MDGs both at mRNA and protein levels. Further, docking results showed lower binding energy for SOCs with MDGs. Subsequently, the validation of these results was carried out using datasets downloaded from gene expression omnibus (GEO). Interestingly, single-cell RNA sequence analysis revealed that gene expression of SOCs is remarkably associated with the MDGs in both cancer and fibroblast cells.

## Materials and Methods

The methodology of the overall study have been represented in the form of graphical abstract (Supplementary Figure 1).

### Gene Ontology Analysis

As a preliminary analysis to show the role of SOCs in signaling pathways we conducted functional enrichment analysis (FEA) to annotate gene ontology (GO) including biological processes (BP), cellular components (CC), molecular function (MF), and pathway enrichment analysis using the Kyoto Encyclopedia of Genes and Genomics (KEGG: http://www.genome.jp) (Kanehisa and Goto, 2000). The MF which showed the highest score for SOCs was visualized using gProfiler (Raudvere et al., 2019). The molecular function with significant *p* adj values are shown. These functions are assigned based on either the experiment or Sequence Model (ISM) or Sequence Alignment (ISA) or Sequence Orthology (ISO) or Sequence or structural similarity (ISS) or Genomic context (IGC) or Biological aspect of ancestor (IBA) or Rapid divergence (IRD) or Reviewed computational analysis (RCA) or Electronic annotation (IEA). The disease ontology analysis was conducted to understand the involvement of SOCs in various cancers. The analysis and visualization for disease ontology were performed using the clusterProfiler package developed by Bioconductor for R statistical environment (Yu et al., 2012; Wu et al., 2022). The adjusted *p*-value of less than 0.05 are considered to be significant.

### The Cancer Genome Atlas (TCGA) Analysis

In the next step, we carried out the TCGA analysis to understand the clinical relevance of SOCs in head and neck cancers. The HiSeqV2 TCGA level 3 gene expression data was downloaded using TCGA biolinks version 2.15.1 developed for R statistical environment (Mounir et al., 2019). The data contained 546 tumor samples and 44 normal samples. Correlation analysis was conducted using Corrplot and ggplot2 packages (Wickham et al., 2016; Wei et al., 2017) and survival analysis was performed using Kaplan-Meier plotter (http://kmplot.com) (Lánczky and Győrffy, 2021; Nagy et al., 2021). The significance and hazard ratio are shown. Further, we conducted mutational analysis to determine the genomic plasticity of SOCs in head neck cancers. Mutations in potential genes encoding SOCs across the TCGA fire hose dataset for HNSC (*n* = 504) are represented as oncoprint plot downloaded from http://cbioportal.org (Cerami et al., 2012). The row of the plot indicates a gene and column indicates the tumor sample.

### Expression Array Data

To further validate the results, we analysed the various expression datasets. Expression profiling dataset GSE171898 contained a total of 323 samples including 208 OSCC tissues from patients treated at Washington University St. Louis and 115 OSCC tissues from patients treated at Vanderbilt University. Illumina Hiseq 3,000 expression profile array data for these samples are available at https://www.ncbi.nlm.nih.gov/geo/query/acc.cgi?acc=GSE171898. The patients were stratified based on the HPV data. The platform used for the GSE17898 expression profiling array was GPL21290 and the spot ID is available at https://www.ncbi.nlm.nih.gov/geo/query/acc.cgi?acc=GPL21290 (Liu et al., 2020).

Single-cell RNA-Seq analysis of head and neck cancer data (GSE103322) were downloaded from the NCBI GEO (Gene Expression Omnibus) database (https://www.ncbi.nlm.nih.gov/geo/). The data consists of an expression profile of 5,902 single cells from 18 patients. The platform used for the GSE103322 expression profiling array was GPL18573 and the spot ID is available at https://www.ncbi.nlm.nih.gov/geo/query/acc.cgi?acc=GPL18573 (Puram et al., 2017).

### Store-Operated Calcium Channels (SOCs) in Mitochondrial Dynamics

The reorganization of ER and Ca^2+^ ion transfer have been implicated in the mitochondrial fission and apoptosis of cancer cells (Yedida et al., 2019). Hence, we next analsyed the relevance of SOCs with genes involved in mitochondrial dynamics. The correlation between expression of genes involved in SOCs and genes involved in mitochondrial genes for all HNSC patients, and HPV positive and negative HNSC patients were downloaded using Timer 2.0 (http://timer.comp-genomics.org/) (Li et al., 2020). The data was visualized using the heatmap function of Graphpad Prism software version 9.2.1. Subsequently, the SOC protein structures and proteins involved in mitochondrial dynamics were downloaded from the protein data bank (Berman et al., 2000). Protein structures for SOCs—STIM1:PDB ID-6YEL (Rathner et al., 2021); STIM2:PDB ID-2L5Y (Zheng et al., 2011); ORAI1:PDB ID-4EHQ (Liu et al., 2012); TRPC5:PDB ID-6YSN (Wright et al., 2020) and MDGs-DNM1L:PDB ID-3W6N (Kishida and Sugio, 2013); MFN1:PDB ID-5GOF (Cao et al., 2017); MFN2:PDB ID-6JFK (Li et al., 2019); OPA1:PDB ID-6JTG (Yu et al., 2020) and FIS1:PDB ID-1PC2 (Suzuki et al., 2003) were downloaded. The docking was performed using Clust Pro version 2.0 (https://cluspro.bu.edu/login.php) (Kozakov et al., 2013; Kozakov et al., 2017; Vajda et al., 2017; Desta et al., 2020) and binding energy was calculated using PRODIGY (https://wenmr.science.uu.nl/prodigy/) (Vangone and Bonvin, 2015; Xue et al., 2016; Vangone et al., 2019). Gene regulatory network was analyzed using Geneck (https://lce.biohpc.swmed.edu/geneck/) (Zhang et al., 2019) and protein-protein interaction network was obtained from string database (https://string-db.org/) (Szklarczyk et al., 2019; Szklarczyk et al., 2021).

### Clinical Proteomic Tumor Analysis Consortium (CPTAC) Data Analysis

Since, the protein expression is critical for the signaling pathways and physiological responses, we next analysed the proteomic and phopshoproteomic levels of SOCs in HNSC. The CPTAC data for proteomics and phosphoproteomics with clinical data for HNSC was downloaded using Python version 3.0 (Huang et al., 2021). The bar plots were plotted using Graphpad Prism version 9.2.1.

### Histology Analysis

The histopathology images for SOCs for HNSC were obtained from Human Protein atlas (https://www.proteinatlas.org/) (Uhlén et al., 2015) that is not active.

### Correlation Analysis

The correlation analysis for SOCs and MDGs gene expression and protein expression were performed using Corrplot package for the R programming (Wei et al., 2017).

### Statistical Analysis

Correlation between different parameters was calculated by Pearson correlation analysis. Differences between the groups were evaluated using nonparametric t-test. *p*-value < 0.05 was considered to be significant for all the TCGA, CPTAC, and GEO datasets analysis. Statistical analysis was performed using R programming version 3.6.1.

## Results

In the present study, we analyzed the expression of SOCs mRNA, proteins, and phosphoproteins of HNSC by *in silico* approaches. In addition, we have also shown the mutations in these genes to understand the effect of genetic alterations on their expression in HNSC patients. Next, we cohered the gene and protein expression levels of SOCs versus MDGs and correlated their expression with the survival risk of HNSC patients. Further, we performed docking to understand the binding efficiency of SOCE proteins with MDGs. To our knowledge, this is the first comprehensive study that links the SOCs gene expression to MDGs and potential risk of early death in HNSC.

### Differential Expression of Store-Operated Calcium Channel Entry in Head and Neck Squamous Cell Cancer

**FIGURE 1:**
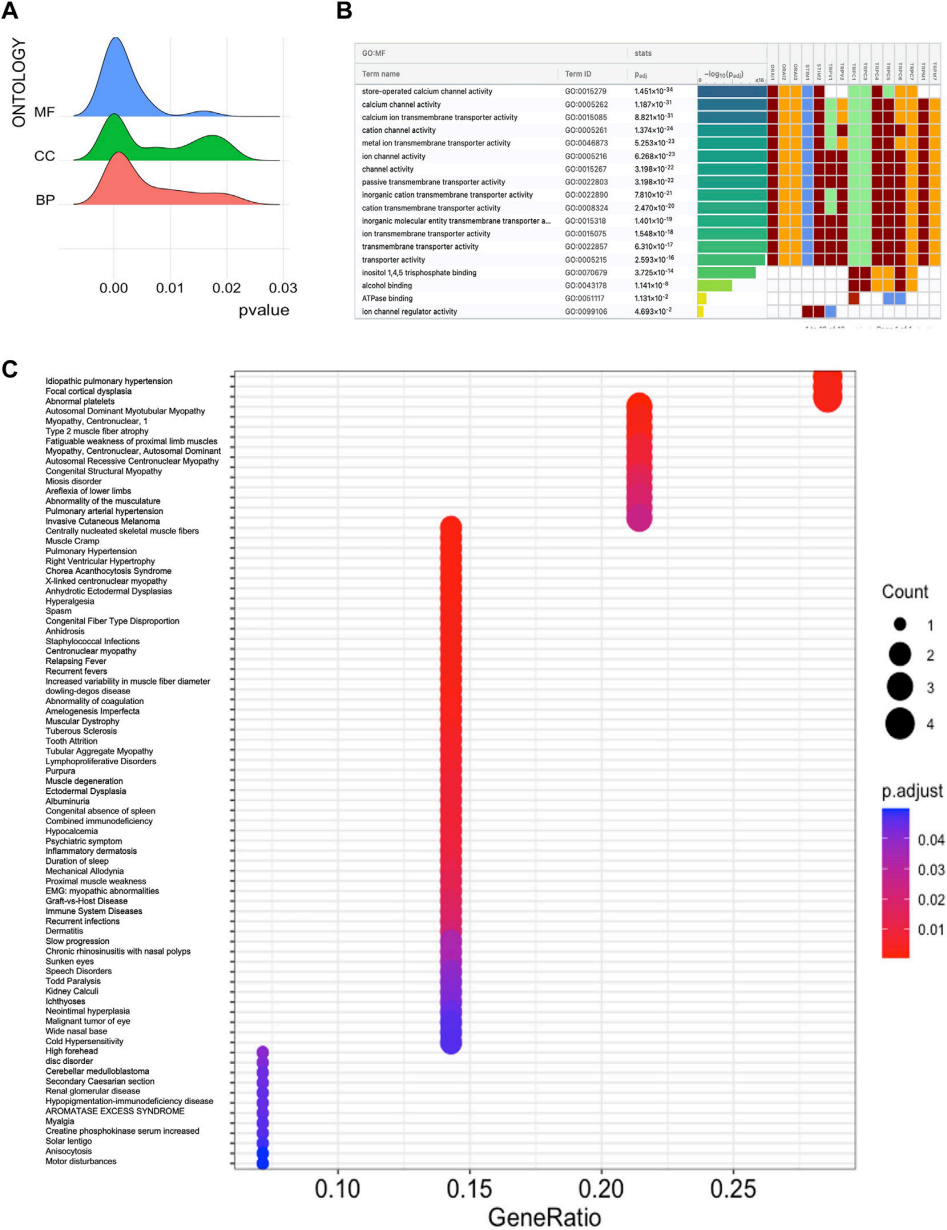
Molecular function and disease ontology of SOCs. **(A)** Ridge plot showing the predominant Gene ontology analysis using ClusterProfile R package. Inset- MF: Molecular function (blue); CC: Cellular component (green); BP: Biological processes (orange) **(B)** Gene ontology analysis using g:Profiler. Molecular function (MF) which showed significant adjusted *p*-value of SOCs is showed. Cumulative hypergeometric padj values are used to dentify the most significant molecular functions. The −log_10_padj column is colored according to the rank with highest being purple to lowest being yellow. The color for each gene is given as follows: red for inferred from the experiment; brown for Sequence Model (ISM), Sequence Alignment (ISA), Sequence Orthology (ISO) or Sequence or structural similarity (ISS), Genomic context (IGC) or Biological aspect of ancestor (IBA), Rapid divergence (IRD); blue for Reviewed computational analysis (RCA), Electronic annotation (IEA); green for traceable author or non traceable author or inferred by curator **(C)** Disease enrichment analysis of SOCs using ClusterProfile R package. The enriched diseases with *p* value less than 0.05 are shown. The dots represent the enrichment of genes with red color being high enrichment and blue being low enrichment. The size of the dot represent the count of each term in the particular row.

**FIGURE 2:**
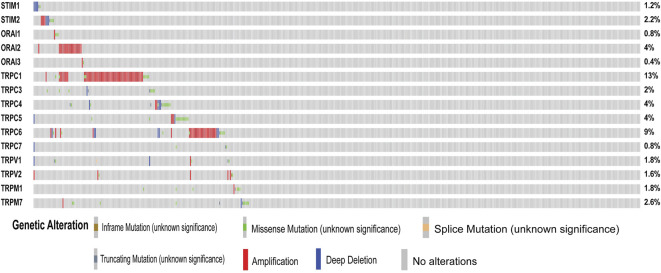
Mutations in SOCs. The mutations in genes associated with SOCs were analysed across the TCGA fire hose (*n* = 504) datasets using Cbioportal. Oncoprint plot was downloaded from the Cbioportal website. The color for different genetic alterations are mentioned at the bottom. Each row in the plot represents a gene and column represents a tumor sample. The percentage of reported mutation rate across the tumor samples (*n* = 504) are represented on the right-hand side.

**FIGURE 3:**
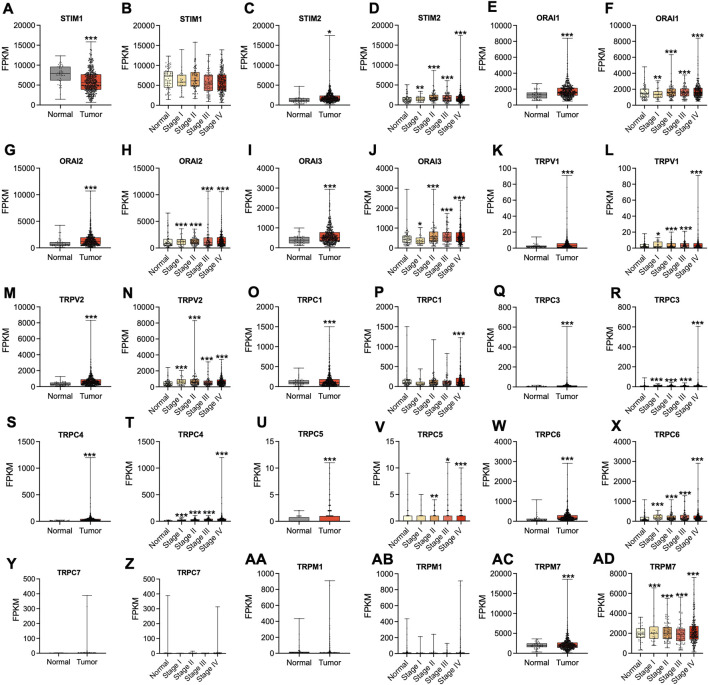
Altered mRNA expression of SOCs in HNSC. The Hiseq2 gene expression and clinical details of TCGA-HNSC data was downloaded from the TCGA biolinks–Bioconductor package for R statistical environment. The expression levels of SOCs (represented as fragments per kilobase per million mapped fragments-FPKM) in normal versus tumor **(A,C,E,G,I,K,M,O,Q,S,U,W, Y, AA, AC)** and different stages- stage I, stage II, stage III, and stage IV of HNSC **(B,D,F,H,J,L,N,P,R,T,V,X, Z, AB, AD)** are plotted. The statistical significance is represented as *p < 0.05, **p < 0.01, ***p < 0.0001.

In the first step, GO and KEGG pathway analysis was carried out for fifteen SOCs genes- STIM1, STIM2, ORAI1, ORAI2, ORAI3, TRPC1, TRPC3, TRPC4, TRPC5, TRPC6, TRPC7, TRPV1, TRPV2, TRPM1, and TRPM7 using clusterProfiler to understand their function in MF, CC, and BP. The genes were enriched mainly for MF (Figure 1A). The molecular function enrichment using gProfiler identified SOCs genes involvement in inositol binding activity and ATPase binding activity which are crucial for cell signaling and regulation of cellular functions apart from the usual calcium transporter, ion transporter, and transmembrane transporter activities of SOCs (Figure 1B). The disease enrichment analysis using ClusterProfiler showed that these genes are involved in pulmonary hypertension, disorders related to muscles, lymphoproliferative disorders, malignant eye tumors, cerebellar medulloblastoma, immune system diseases, and inflammatory disorders (Figure 1C). This indicated that SOCs play critical role in tumor development and progression. Further, we analysed for the mutation in SOCs across TCGA fire hose datasets containing 504 samples for HNSC. The mutation analysis showed 1.2% mutation in STIM1 (6/504), 2.2% mutation in STIM2 (11/504), 0.8% mutation in ORAI1 (4/504), 4% mutation in ORAI2 (20/504), 0.4% mutation in ORAI3 (2/504), 13% in TRPC1 (64/504), 2% mutation in TRPC3 (10/504), 4% mutation in TRPC4 (20/504), 4% mutation in TRPC5 (20/504), 9% mutation in TRPC6 (43/504), 0.8% mutation in TRPC7 (4/504), 1.8% mutation in TRPV1 (9/504), 1.6% mutation in TRPV2 (8/504), 1.8% mutation in TRPM1 (9/504), and 2.6% mutation in TRPM7 (13/504). This low percentage of mutations indicated that mutation is solely not responsible for the aberration in the SOCs expression (Figure 2). Hence, we further analysed the mRNA expression of SOCs. The mRNA expression of SOCs genes across TCGA-HNSC data was downloaded from the TCGA biolinks R package. The data showed significant upregulation of STIM2 (*p* < 0.05), ORAI1 (*p* < 0.001), ORAI2 (*p* < 0.001), ORAI3 (*p* < 0.001), TRPC1 (*p* < 0.001), TRPC3 (*p* < 0.001), TRPC4 (*p* < 0.001), TRPC5 (*p* < 0.001), TRPC6 (*p* < 0.001), TRPV1 (*p* < 0.001), TRPV2 (*p* < 0.001), and TRPM7 (*p* < 0.001) and downregulation of STIM1 (*p* < 0.001) and TRPM1 (*p* > 0.05) were observed across HNSC samples. The expression level of TRPC7 was found to be low in both controls and HNSC patients and was insignificant. We also conducted the stage-wise expression analysis of these genes in HNSC. The data showed significant upregulation of STIM2 (Normal vs. stage I: *p* < 0.01; Normal vs. stage II: *p* < 0.001; Normal vs. stage III: *p* < 0.001; Normal vs. stage IV: *p* < 0.001), ORAI1 (Normal vs. stage I: *p* < 0.01; Normal vs. stage II: *p* < 0.001; Normal vs. stage III: *p* < 0.001; Normal vs. stage IV: *p* < 0.001), ORAI2 (Normal vs. stage I: *p* < 0.001; Normal vs. stage II: *p* < 0.001; Normal vs. stage III: *p* < 0.001; Normal vs. stage IV: *p* < 0.001), ORAI3 (Normal vs. stage I: *p* < 0.05; Normal vs. stage II: *p* < 0.001; Normal vs. stage III: *p* < 0.001; Normal vs. stage IV: *p* < 0.001), TRPC3 (Normal vs. stage I: *p* < 0.001; Normal vs. stage II: *p* < 0.001; Normal vs. stage III: *p* < 0.001; Normal vs. stage IV: *p* < 0.001), TRPC4 (Normal vs. stage I: *p* < 0.001; Normal vs. stage II: *p* < 0.001; Normal vs. stage III: *p* < 0.001; Normal vs. stage IV: *p* < 0.001), TRPC5 (Normal vs. stage I: *p* > 0.05; Normal vs. stage II: *p* < 0.01; Normal vs. stage III: *p* < 0.05; Normal vs. stage IV: *p* < 0.001), TRPC6 (Normal vs. stage I: *p* < 0.001; Normal vs. stage II: *p* < 0.001; Normal vs. stage III: *p* < 0.001; Normal vs. stage IV: *p* < 0.001), TRPV1 (Normal vs. stage I: *p* < 0.05; Normal vs. stage II: *p* < 0.001; Normal vs. stage III: *p* < 0.001; Normal vs. stage IV: *p* < 0.001), TRPV2 (Normal vs. stage I: *p* < 0.001; Normal vs. stage II: *p* < 0.001; Normal vs. stage III: *p* < 0.001; Normal vs. stage IV: *p* < 0.001), and TRPM7 (Normal vs. stage I: *p* < 0.001; Normal vs. stage II: *p* < 0.001; Normal vs. stage III: *p* < 0.001; Normal vs. stage IV: *p* < 0.001) across all the stages of HNSC. TRPC1 (*p* < 0.001) level was found to be significantly altered in stage IV disease. STIM1, TRPC7, and TRPM1 were found to be non-significant across the stages of HNSC compared to controls (Figures 3A-AD). These results indicated that SOCs are differentially expressed in various stages of head and neck cancers and could be a potential biomarker of HNSC.

### Correlation of Store-Operated Calcium Channels Gene Expression and Genes Involved in Mitochondrial Dynamics (MDGs)

**FIGURE 4:**
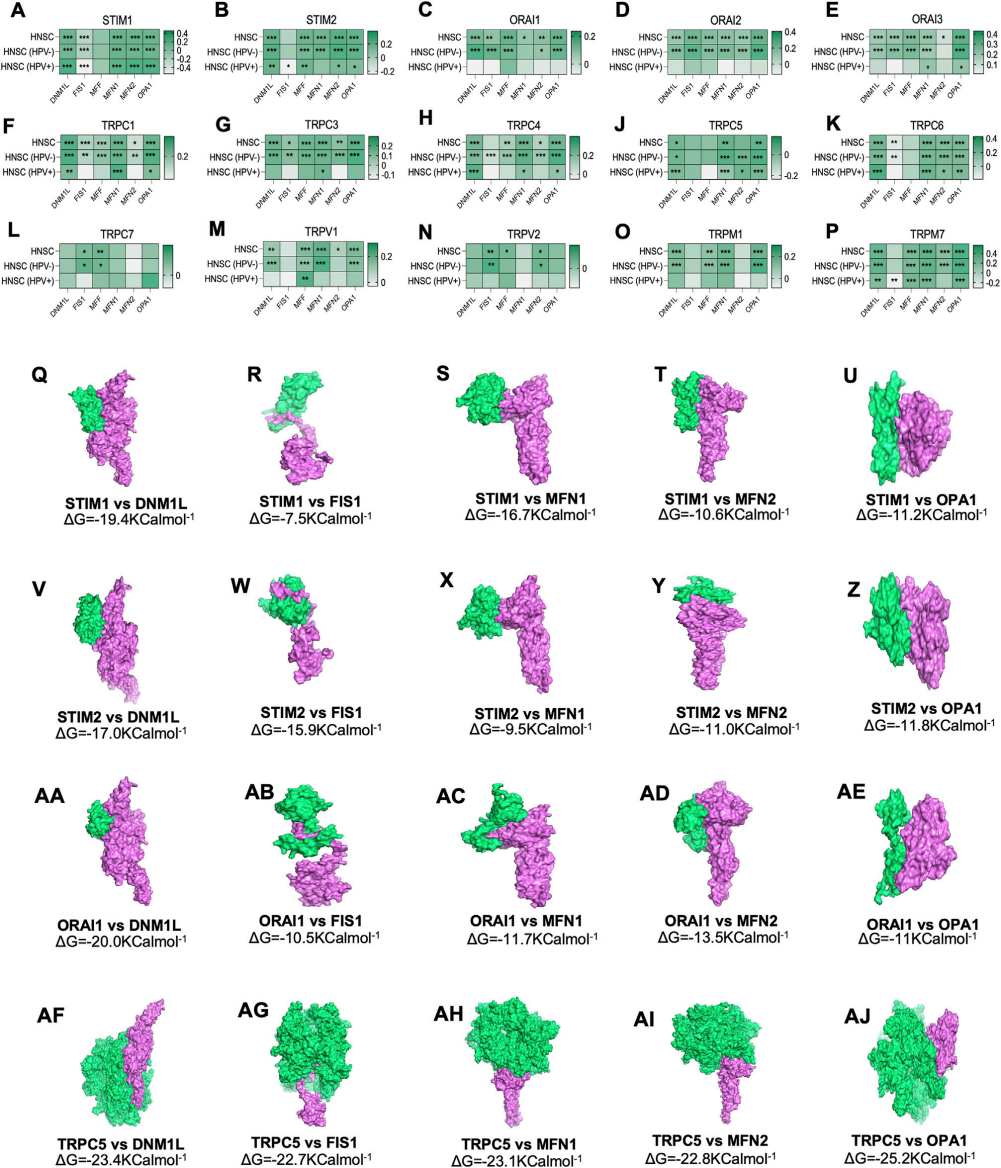
Altered mRNA expression of SOCs are correlated with the expression of MDGs in HNSC. **(A–P)** The Hiseq2 gene expression TCGA-HNSC data was downloaded. The expression levels of SOCs are compared with the expression of MDGs using Timer 2.0 tool. The statistical significance is represented in asterisk. *p < 0.05, **<0.01, ***p < 0.001. **(Q–AJ)** Protein structures for SOCs- STIM1 (PDB ID-6YEL), STIM2 (PDB ID-2L5Y), ORAI1 (PDB ID-4EHQ), TRPC5 (PDB ID-6YSN)- and MDGs-DNM1L (PDB ID-3W6N), MFN1 (PDB ID-5GOF), MFN2 (PDB ID-6JFK), OPA1(PDB ID-6JTG) and FIS1 (PDB ID-1PC2) were downloaded in the. pdb format from protein data bank and docked using ClustPro tool. The protein in green represents SOCs and pink represents MDGs. Prodigy was used to calculate ΔG.

**FIGURE 5:**
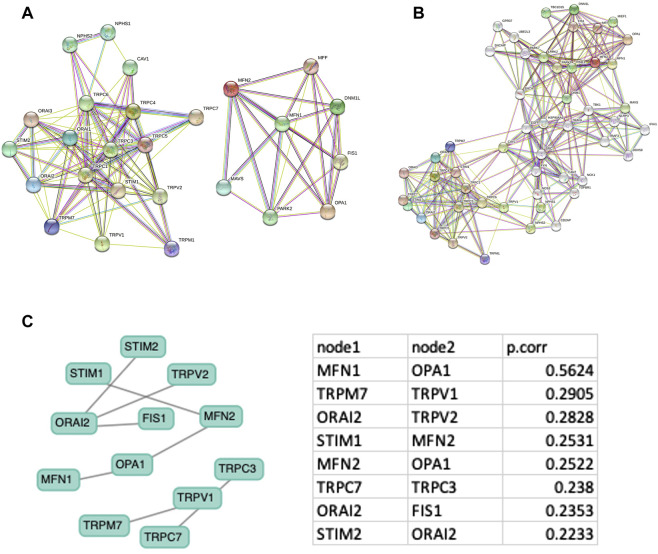
Protein-protein network analysis. Protein-protein interaction network with minimum node **(A)** and increased node **(B)** were downloaded from STRING database **(C)** Gene interaction network is downloaded from GeNeck tool and Pearson correlation for each node is shown.

The expression of SOCs genes and mitochondrial dynamics regulatory genes- DNM1L, FIS1, MFF, MFN1, MFN2, OPA1- across TCGA-HNSC data were conducted using Timer 2.0 webtool. The expression of SOCs genes was found to be remarkably associated with the expression of DNM1L, FIS1, MFN1, MFN2, and OPA1. Similarly, expression of SOCs genes with MDGs in HPV positive and HPV negative HNSC patient samples are also shown (Figures 4A–P). Next, we downloaded available protein structures for SOCs (STIM1, STIM2, ORAI1, TRPC5) and mitochondrial fission and fusion regulatory genes (DNM1L, FIS1, MFF, MFN1, MFN2, and OPA1) from the protein data bank. Protein-protein docking using ClustPro and Prodigy showed high negative binding energy for these proteins—STIM1 vs. DNM1L:ΔG = −19.4KCalmol^−1^, STIM1 vs. FIS1:ΔG = −7.5KCalmol^−1^, STIM1 vs. MFN1:ΔG = −16.7KCalmol^−1^, STIM1 vs. MFN2:ΔG = −10.6KCalmol^−1^, STIM1 vs. OPA1:ΔG = −11.2KCalmol^−1^; STIM2 vs. DNM1L: ΔG = −17.0KCalmol^−1^; STIM2 vs. FIS1:ΔG = −15.9KCalmol^−1^, STIM2 vs. MFN1:ΔG = −9.5KCalmol^−1^, STIM2 vs. MFN2:ΔG = −11.0KCalmol^−1^; STIM2 vs. OPA1:ΔG = −11.8KCalmol^−1^; ORAI1 vs. DNM1L:ΔG = −20.0KCalmol^−1^, ORAI1 vs. FIS1:ΔG = −10.5KCalmol^−1^, ORAI1 vs. MFN1:ΔG = −11.7KCalmol^−1^, ORAI1 vs. MFN2:ΔG = −13.5KCalmol^−1^, ORAI vs. OPA1:ΔG = −11KCalmol^−1^; TRPC5 vs. DNM1L:ΔG = −23.4KCalmol^−1^, TRPC5 vs. FIS1:ΔG = −22.7KCalmol^−1^, TRPC5 vs. MFN1:ΔG = −23.1KCalmol^−1^, TRPC5 vs. MFN2:ΔG = −22.8KCalmol^−1^, TRPC5 vs. OPA1:ΔG = −25.2KCalmol^−1^ indicating higher chances of binding of SOCs with proteins involved in mitochondrial dynamics (Figures 4Q–AJ). However, STRING analysis showed no known direct link between SOCs and MDGs (Figures 5A–C).

### Survival Analysis

**FIGURE 6:**
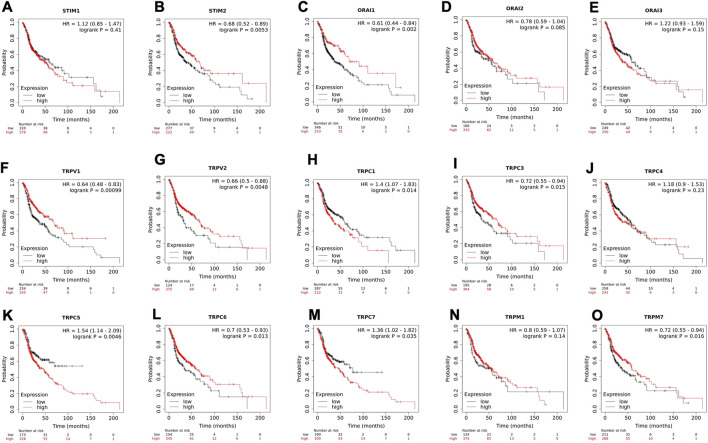
The altered expression of SOCs resulted in poor survival of HNSC patients. **(A-O)** Kaplan-meier plot for all the SOCs expression in HNSC patients was downloaded from KMplotter. The probability of survival for high and low expression are shown. Inset- Hazard ratio (HR) with 95% confidence interval and logrank *p* value are mentioned. The number of patients at risk during the time interval is given below the graph.

**FIGURE 7:**
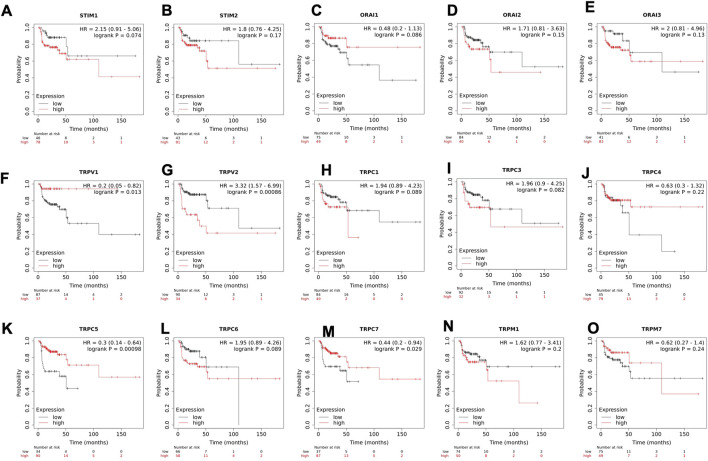
Altered mRNA expression of SOCs lead to poor relapse survival of HNSC patients. **(A-O)** Kaplan-meier plot for relapse free survival of head and neck cancer patients with altered expression of SOCs are shown. The survival probability versus time for high and low expression of SOCs are shown. Inset- Hazard ratio (HR) with 95% confidence interval and logrank *p* value are shown. The number of patients at risk during each time interval for both high and low expression of SOCs is represented below the graph.

**FIGURE 8:**
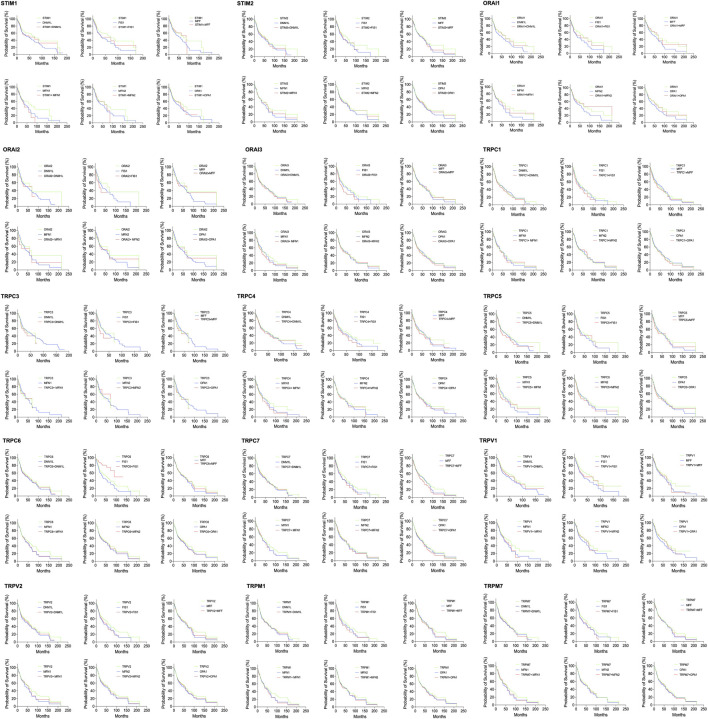
Survival analysis among Head neck cancer patients expressing altered levels of SOCs in conjunction with MDGs. The survival data from TCGA was downloaded using TCGA biolinks package built for R statistical environment. The survival data for expression levels of each SOCs in conjunction with MDGs is plotted.

To understand the clinical relevance of these SOCs genes, the correlation between gene expression versus overall survival and relapse-free survival of HNSC patients were explored. Kaplan-Meier plotter was used to analyze overall survival analysis across TCGA-HNSC samples. The expression of STIM2, ORAI1, TRPV1, TRPV2, TRPC1, TRPC3, TRPC5, TRPC6, TRPC7, and TRPM7 were found to be significantly (*p* < 0.05) associated with HNSC patient survival (Figures 6A–O). The effect of expression of SOCs along with MDGs on survival of HNSC was visualized. Similar survival analysis was performed for relapse-free survival across TCGA data sets and are shown in Figures 7A–O. Next, the survival data were downloaded using TCGA biolinks via R programming. We analysed the survival probability for patients expressing SOCs in conjunction with the MDGs and found that SOCs along with MDGs are potential diagnostic and prognostic markers of HNSC (Figure 8).

### Expression of Store-Operated Calcium Channels and Their Correlation With Mitochondrial Dynamics Among Clinical Proteomic Tumor Analysis Consortium (CPTAC) and Gene Expression Omnibus (GEO) Datasets

**FIGURE 9:**
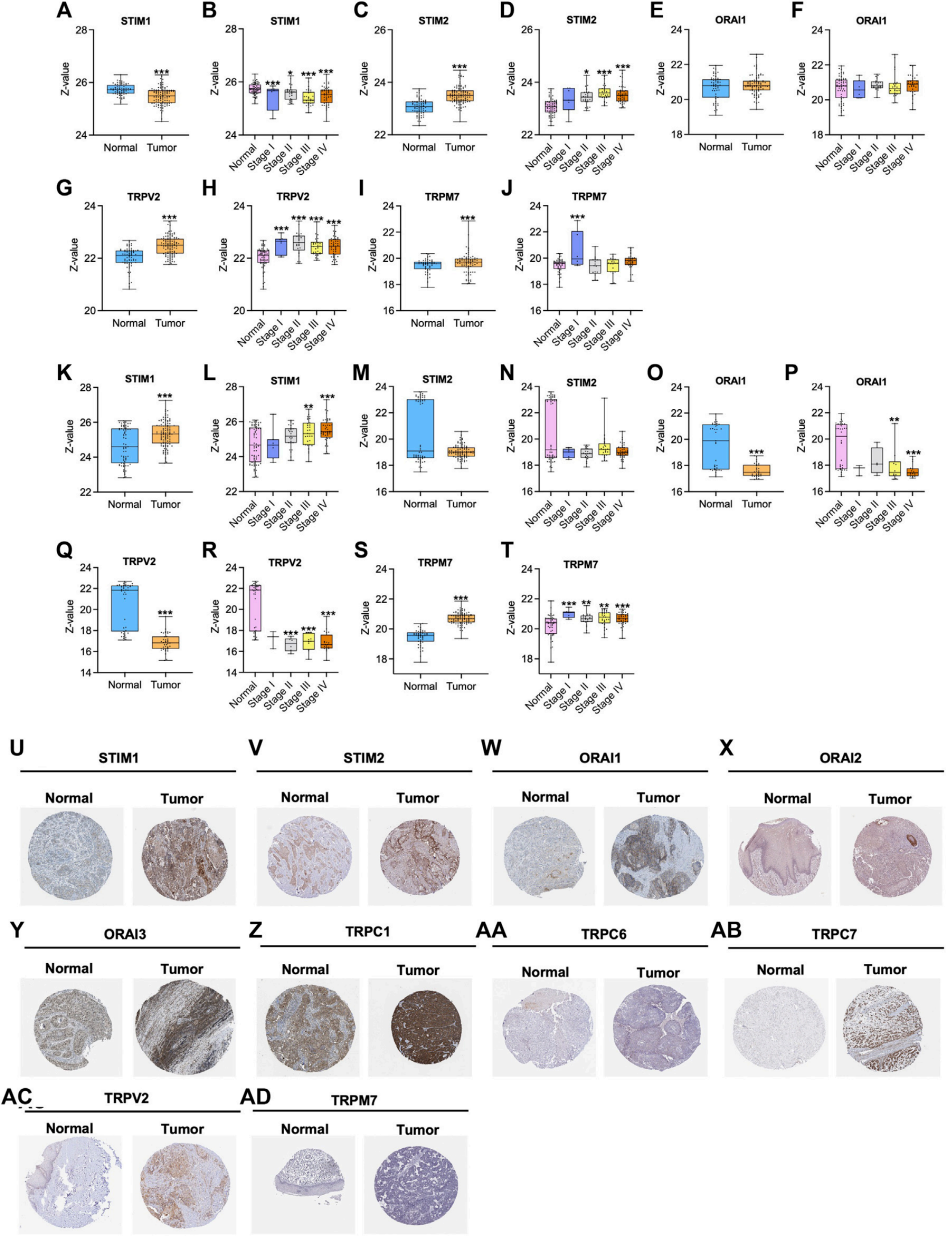
Altered protein expression of SOCs in HNSC. **(A–J)** The proteomics and **(K–T)** phosphoproteomics expression was downloaded from CPTAC-HNSC database using Python 3.0. The protein and phosphoprotein expression of SOCs were plotted. The phosphoprotein sites which are considered to plot this graph include: S257 for STIM1; S261 for STIM2; T295 for ORAI1; S751 for TRPV2; S1477 for TRPM7. Asterisk represents *p < 0.05, **<0.01, ***p < 0.001 the statistical significance. **(U–AD)** Histopathology slides of tissue microarray were downloaded from Human Protein Atlas and the expression of the SOCs are presented.

**FIGURE 10:**
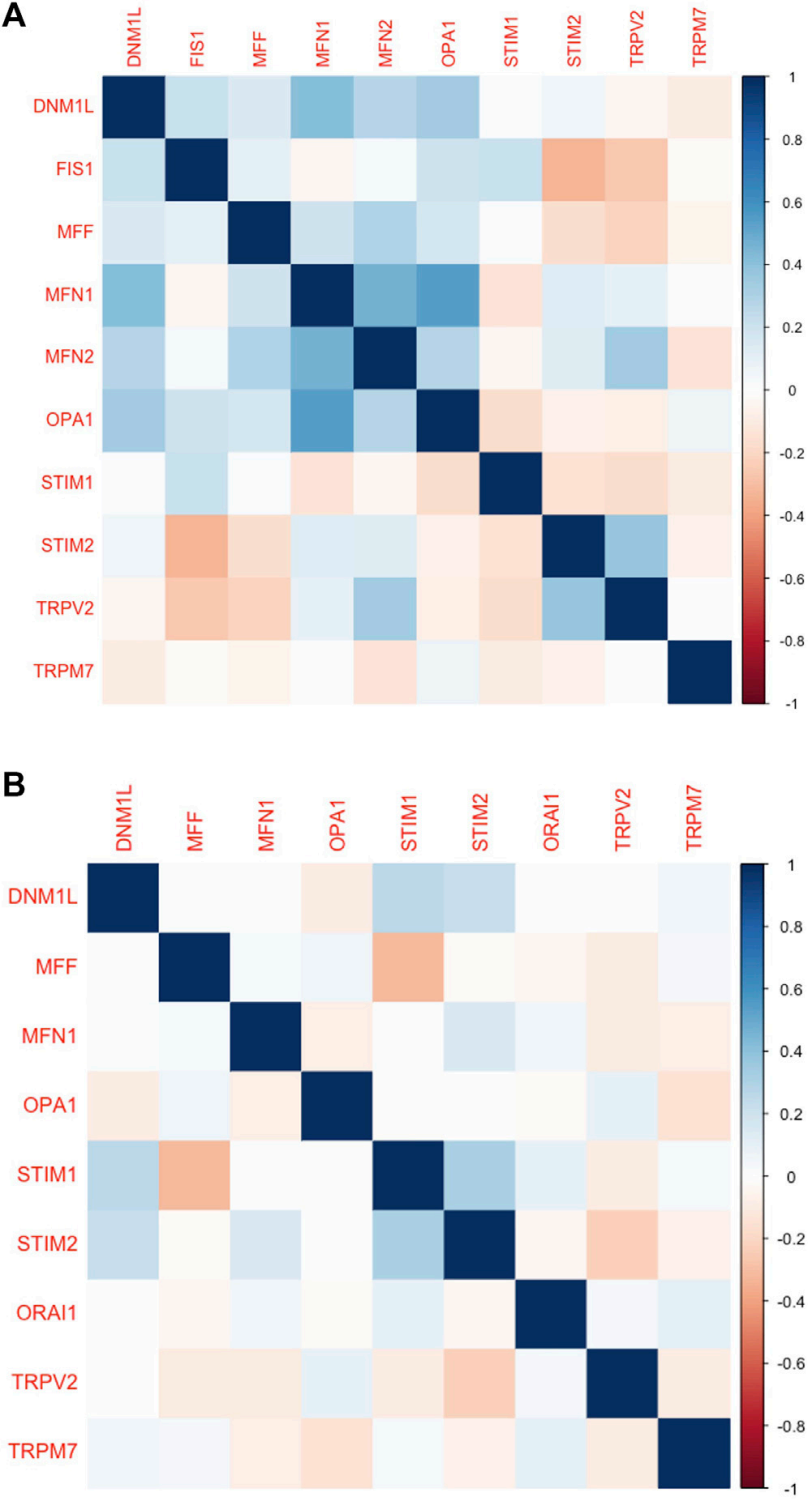
Protein levels of SOCs correlated with the MDGs in HNSC. **(A)** The proteomics and **(B)** phospho-proteomics expression CPTAC-HNSC data was downloaded using Python 3.0. The correlation was calculated and plotted using Corr plot package for R programming. The markings on right hand side indicates the color code for correlation coefficient.

**FIGURE 11:**
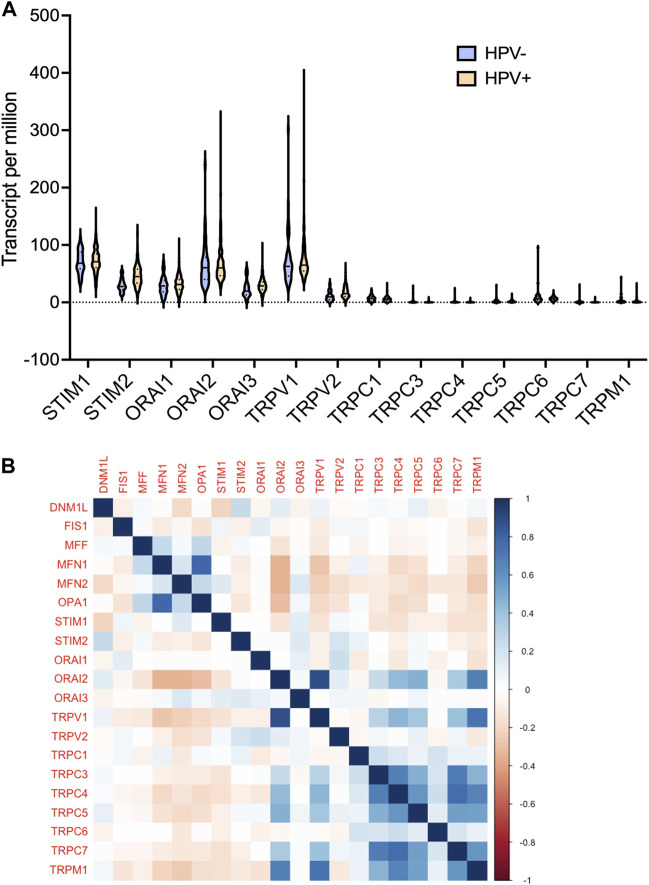
mRNA expression levels of SOCs were correlated with the expression of MDGs in both HPV positive and negative HNSCs. The expression data from GSE17898 was downloaded from GEO datasets consisting total of 323 samples **(A)** The levels of SOCs are plotted using Graphpad prism software and **(B)** correlation analysis was performed and plotted using Corr Plot package for R statistical environment. The gradation on the right side denote the correlation coefficient.

Furthermore, we analysed the protein expression of SOCs and MDGs in HNSC. The proteomics, phosphoproteomics, and clinical data were obtained for CPTAC-HNSC using Python version 3.0. The protein expression was available only for STIM1, STIM2, ORAI1, TRPV2, and TRPM7 in CPTAC dataset. This might be due to the spatio-temporal expression of proteins which is unrelated to the mRNA expression of genes. The available data were analyzed and visualized. The protein (Figures 9A–J) and phosphoprotein levels (Figures 9K–T) showed significant alteration of STIM1, STIM2, ORAI1, TRPV2, and TRPM7 in HNSC patients both overall and stage-wise compared to control samples. The histopathology slides downloaded from the human protein atlas also showed similar results (Figures 9U–AD). Additionally, the correlation of SOCs proteins and phosphoproteins with MD proteins were analyzed using Corr Plot package in R statistical environment (Figures 10A,B). In addition, the dataset GSE17898 consisting of normalized expression data from a total of 323 HNSC samples were downloaded and analyzed. The data showed remarkable alteration of SOCs in both HPV positive and HPV negative samples (Figure 11A). The correlation analysis showed a significant association among SOCs and MDGs expression which is in accordance with our TCGA and CPTAC analysis (Figure 11B).

### Single-Cell Gene Expression Analysis of Store-Operated Calcium Channels and Their Correlation With Mitochondrial Dynamics

**FIGURE 12:**
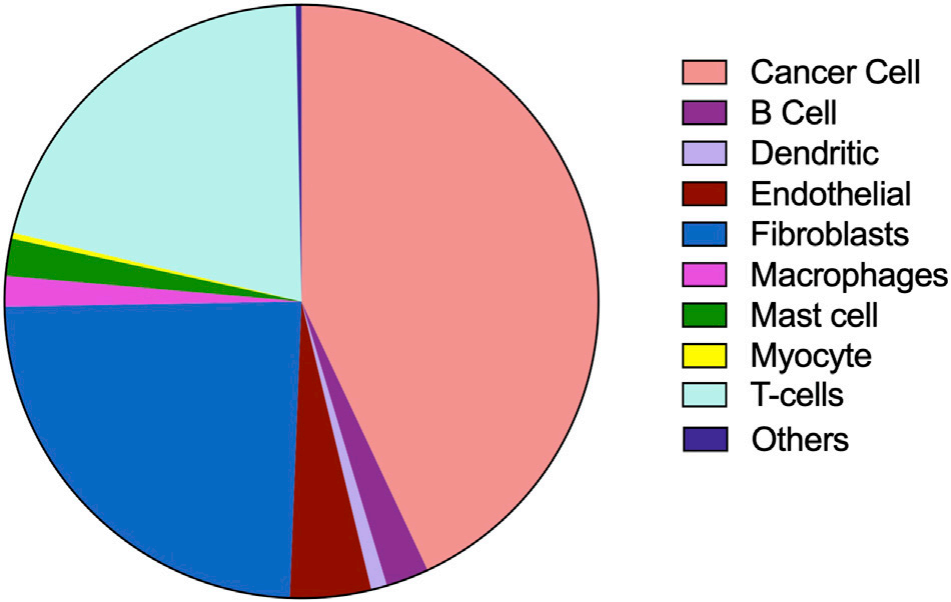
Composition of different cell types in tumor microenvironment analysed using the dataset GSE103322. The percentage composition of cancer cells, B cells, dendritic cells, endothelial cells, fibroblasts, macrophages, mast cells, myocytes, T cells and other cells are visualized using pie-chart representation.

**FIGURE 13:**
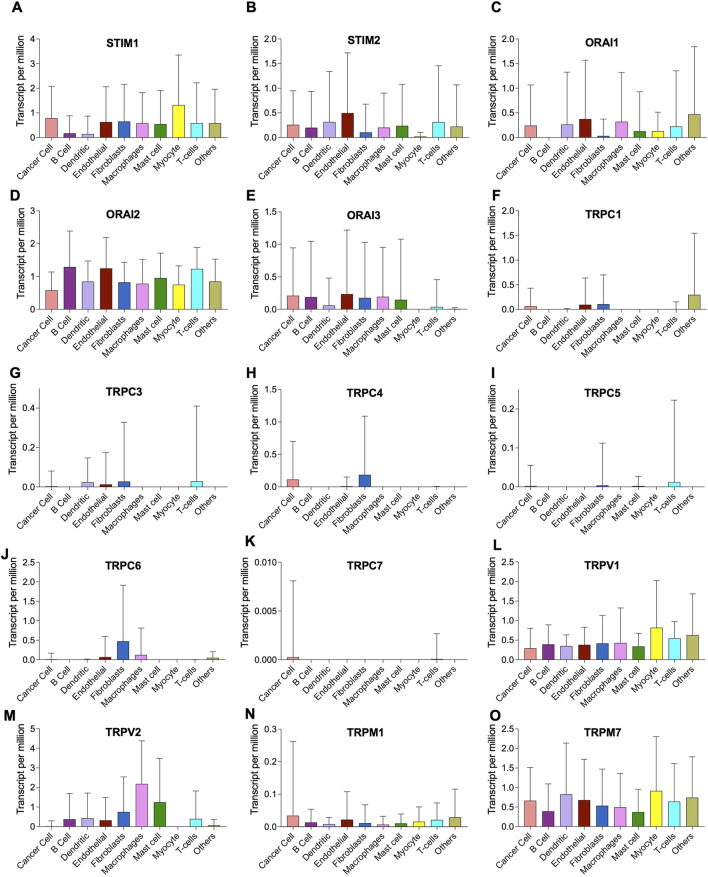
Heterogenous expression of SOCs in tumor microenvironment. **(A-O)** The expression data from GSE103322 comprising the data of 5,902 cells from 18 head and neck cancer tissues was downloaded from NCBI GEO website. The data comprised of different cell populations including cancer cells, B cells, dendritic cells, endothelial cells, fibroblasts, macrophages, mast cells, myocytes, T cells and other cells The expression levels of each SOCs in these different cell types are plotted using Graphpad prism software version 9.2.1.

**FIGURE 14:**
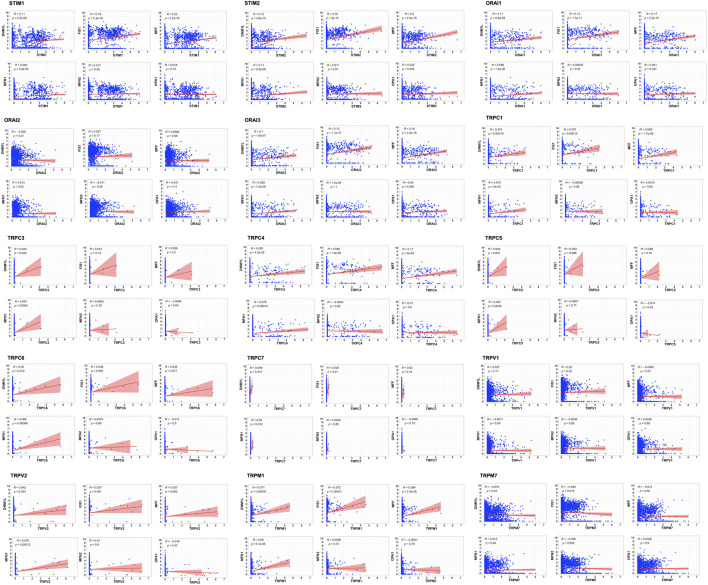
Correlation of SOCs expression with MDGs in cancer cells across dataset GSE103322. The correlation between each SOCs expression (STIM1, STIM2, ORAI1, ORAI2, ORAI3, TRPC1, TRPC3, TRPC4, TRPC5, TRPC6, TRPC7, TRPV1, TRPV2, TRPM1, and TRPM7) versus MDGs expression (DNM1L, FIS1, MFF, MFN1, MFN2, and OPA1) among the cancer cells are plotted using Corr plot package of R statistical environment. The linear regression line per group is represented as red colored line. Inset- R value (correlation coefficient) and *p* value are mentioned.

**FIGURE 15:**
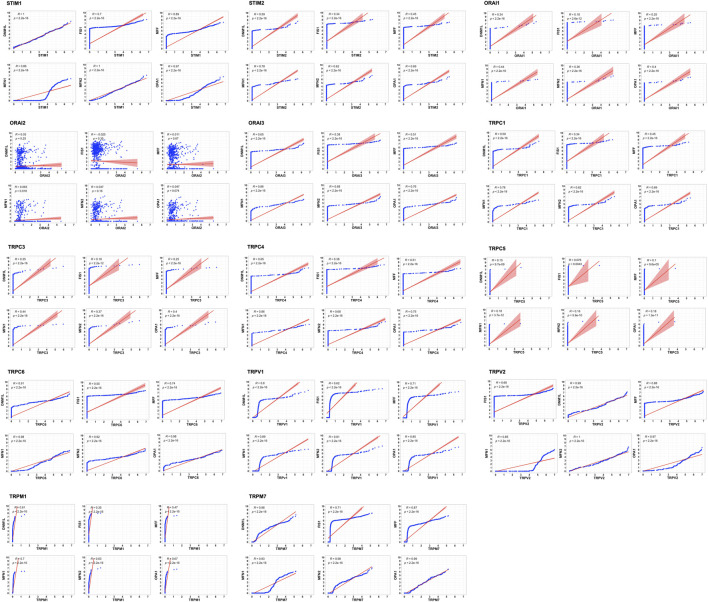
Correlation of SOCs expression with MDGs in fibroblasts across dataset GSE103322. The expression of each SOCs (STIM1, STIM2, ORAI1, ORAI2, ORAI3, TRPC1, TRPC3, TRPC4, TRPC5, TRPC6, TRPV1, TRPV2, TRPM1, and TRPM7) versus expression of each MDGs (DNM1L, FIS1, MFF, MFN1, MFN2, and OPA1) were compared among the fibroblasts and the correlation plots are plotted using Corr plot package of R statistical environment. TRPC7 is not expressed in the fibroblasts and is omitted. Linear regression line is shown in red color. Inset- R value (correlation coefficient) and *p* value are shown.

The tumor microenvironment consists of heterogenous population of cells contributing separately to the proliferation, development, metastasis, and therapeutic resistance (Da Silva-Diz et al., 2018; Stanta and Bonin, 2018; El-Sayes et al., 2021). Hence, we further analyzed single cell dataset for HNSC (GSE130922) downloaded from NCBI GEO datasets. Our analysis showed heterogeneous expression of SOCs in different cells of HNSC tumor tissues (Puram et al., 2017). The data was stratified across 10 different cell types- cancer cells and B cells, dendritic cells, endothelial cells, fibroblasts, macrophages, mast cells, monocytes, T-cells, and others among non-cancer stromal cells. The percentage population of these cells in the dataset are shown in Figure 12. STIM1, STIM2, ORAI1, ORAI2, ORAI3, TRPV1, TRPV2, and TRPM7 were found to be differentially expressed across all cell types in HNSC tumors (Figures 13A-O). Among the immune cells, STIM1 is expressed in all the immune cells with the highest expression in mast cells and lowest in dendritic and B cells. STIM2 and ORAI2 are expressed nearly equally in all the immune cells. ORAI1 levels were found to be nil in B cells. ORAI3 is found to be least expressed in dendritic and T cells. TRPC1, TRPC4, TRPC6, and TRPC7 are almost completely absent in all immune cells types whereas TRPC3 is expressed in T cells and dendritic cells, and TRPC5 is expressed only in T cells. TRPV1, TRPM1, and TRPM7 are almost equally expressed in all types of immune cells. TRPV2 is found to be expressed highly in macrophages. However, expression of ORAI2, TRPC5, and TRPV2 are high in B cells, T cells, and macrophages respectively compared to parenchymal cells. However, other SOCs are enriched in parenchymal cells including cancer cells compared to immune cells. In addition, SOCs were found to be highly expressed in cancer cells and fibroblasts among the parenchymal cells (Figures 13A-O). Further, a significant correlation of SOCs with MDGs were observed in cancer cells (Figure 14) and fibroblasts (Figure 15). The correlation coefficient and *p*-value are represented in the Table 1.

**TABLE 1:** The correlation coefficient and *p*-value for SOCs and MDGs in cancer cells and fibroblasts.

Cell type	SOCs	MDGs	Correlation coefficient	*p*-Value
Cancer cell	STIM1	DNM1L	0.11	2.5 × 10^−8^
		FIS1	0.18	<2.2 × 10^−16^
		MFF	0.22	<2.2 × 10^−16^
		MFN1	0.083	2.6 × 10^−5^
		MFN2	0.017	0.38
		OPA1	0.018	0.37
	STIM2	DNM1L	0.12	4.8 × 10^−10^
		FIS1	0.16	3 × 10^−15^
		MFF	0.2	<2.2 × 10^−16^
		MFN1	0.11	9.6 × 10^−8^
		MFN2	0.011	0.57
		OPA1	0.042	0.035
	ORAI1	DNM1L	0.11	6.8 × 10^−8^
		FIS1	0.13	1.7 × 10^−11^
		MFF	0.17	<2.2 × 10^−16^
		MFN1	0.096	1.4 × 10^−6^
		MFN2	0.00079	0.97
		OPA1	0.041	0.037
	ORAI2	DNM1L	−0.025	0.21
		FIS1	0.027	0.17
		MFF	0.0082	0.68
		MFN1	0.013	0.52
		MFN2	-0.011	0.56
		OPA1	0.031	0.11
	ORAI3	DNM1L	0.1	1.6 × 10^−7^
		FIS1	0.13	1.1 × 10^−10^
		MFF	0.16	2.2 × 10^−16^
		MFN1	0.092	3.3 × 10^−6^
		MFN2	7.3 × 10^−5^	1
		OPA1	0.04	0.046
	TRPC1	DNM1L	0.074	0.00019
		FIS1	0.075	0.00015
		MFF	0.095	1.7 × 10^−6^
		MFN1	0.074	2 × 10^−4^
		MFN2	−0.00095	0.96
		OPA1	0.0015	0.94
	TRPC3	DNM1L	0.043	0.029
		FIS1	0.031	0.12
		MFF	0.026	0.2
		MFN1	0.057	0.0042
		MFN2	0.0062	0.76
		OPA1	−0.0094	0.64
	TRPC4	DNM1L	0.081	4.2 × 10^−5^
		FIS1	0.085	1.9 × 10^−5^
		MFF	0.11	3 × 10^−8^
		MFN1	0.075	0.00014
		MFN2	−0.0043	0.83
		OPA1	0.01	0.6
	TRPC5	DNM1L	0.044	0.027
		FIS1	0.033	0.098
		MFF	0.028	1.6
		MFN1	0.057	0.0039
		MFN2	0.0057	0.77
		OPA1	−0.014	0.49
	TRPC6	DNM1L	0.05	0.012
		FIS1	0.038	0.056
		MFF	0.035	0.077
		MFN1	0.068	0.00066
		MFN2	0.0079	0.69
		OPA1	−0.013	0.5
	TRPC7	DNM1L	0.049	0.14
		FIS1	0.025	0.21
		MFF	0.03	0.14
		MFN1	0.05	0.012
		MFN2	0.0038	0.85
		OPA1	−0.0069	0.73
	TRPV1	DNM1L	0.027	0.17
		FIS1	0.02	0.32
		MFF	−0.0085	0.67
		MFN1	−0.0011	0.95
		MFN2	−0.0036	0.85
		OPA1	0.0028	0.89
	TRPV2	DNM1L	0.042	0.033
		FIS1	0.031	0.06
		MFF	0.037	0.065
		MFN1	0.076	0.00012
		MFN2	0.01	0.6
		OPA1	−0.018	0.37
	TRPM1	DNM1L	0.071	0.00035
		FIS1	0.072	0.00031
		MFF	0.084	2.4 × 10^−5^
		MFN1	0.09	6.1 × 10^−6^
		MFN2	−0.0068	0.73
		OPA1	−0.0061	0.76
	TRPM7	DNM1L	−0.016	0.43
		FIS1	−0.048	0.016
		MFF	−0.013	0.52
		MFN1	0.015	0.46
		MFN2	−0.038	0.055
		OPA1	0.0025	0.9
Fibroblasts	STIM1	DNM1L	1	<2.2 × 10^−16^
		FIS1	0.7	<2.2 × 10^−16^
		MFF	0.89	<2.2 × 10^−16^
		MFN1	0.86	<2.2 × 10^−16^
		MFN2	1	<2.2 × 10^−16^
		OPA1	0.97	<2.2 × 10^−16^
	STIM2	DNM1L	0.59	<2.2 × 10^−16^
		FIS1	0.34	<2.2 × 10^−16^
		MFF	0.45	<2.2 × 10^−16^
		MFN1	0.78	<2.2 × 10^−16^
		MFN2	0.62	<2.2 × 10^−16^
		OPA1	0.69	<2.2 × 10^−16^
	ORAI1	DNM1L	0.34	<2.2 × 10^−16^
		FIS1	0.18	=2.2 × 10^−16^
		MFF	0.25	<2.2 × 10^−16^
		MFN1	0.44	<2.2 × 10^−16^
		MFN2	0.36	<2.2 × 10^−16^
		OPA1	0.4	<2.2 × 10^−16^
	ORAI2	DNM1L	0.03	0.25
		FIS1	-0.025	0.35
		MFF	0.011	0.67
		MFN1	0.063	0.018
		MFN2	0.037	0.16
		OPA1	0.047	0.074
	ORAI3	DNM1L	0.65	<2.2 × 10^−16^
		FIS1	0.38	<2.2 × 10^−16^
		MFF	0.51	<2.2 × 10^−16^
		MFN1	0.86	<2.2 × 10^−16^
		MFN2	0.68	<2.2 × 10^−16^
		OPA1	0.76	<2.2 × 10^−16^
	TRPC1	DNM1L	0.59	<2.2 × 10^−16^
		FIS1	0.34	<2.2 × 10^−16^
		MFF	0.45	<2.2 × 10^−16^
		MFN1	0.78	<2.2 × 10^−16^
		MFN2	0.62	<2.2 × 10^−16^
		OPA1	0.69	<2.2 × 10^−16^
	TRPC3	DNM1L	0.35	<2.2 × 10^−16^
		FIS1	0.18	<2.2 × 10^−16^
		MFF	0.25	<2.2 × 10^−16^
		MFN1	0.44	<2.2 × 10^−16^
		MFN2	0.37	<2.2 × 10^−16^
		OPA1	0.4	<2.2 × 10^−16^
	TRPC4	DNM1L	0.65	<2.2 × 10^−16^
		FIS1	0.38	<2.2 × 10^−16^
		MFF	0.51	<2.2 × 10^−16^
		MFN1	0.86	<2.2 × 10^−16^
		MFN2	0.68	<2.2 × 10^−16^
		OPA1	0.75	<2.2 × 10^−16^
	TRPC5	DNM1L	0.15	9.7 × 10^−9^
		FIS1	0.076	0.0043
		MFF	0.1	<2.2 × 10^−16^
		MFN1	0.18	3.7 × 10^−12^
		MFN2	0.16	5.9 × 10^−10^
		OPA1	0.18	1.3 × 10^−11^
	TRPC6	DNM1L	0.91	<2.2 × 10^−16^
		FIS1	0.55	<2.2 × 10^−16^
		MFF	0.74	<2.2 × 10^−16^
		MFN1	0.98	<2.2 × 10^−16^
		MFN2	0.92	<2.2 × 10^−16^
		OPA1	0.98	<2.2 × 10^−16^
	TRPV1	DNM1L	0.8	<2.2 × 10^−16^
		FIS1	0.62	<2.2 × 10^−16^
		MFF	0.71	<2.2 × 10^−16^
		MFN1	0.89	<2.2 × 10^−16^
		MFN2	0.81	<2.2 × 10^−16^
		OPA1	0.85	<2.2 × 10^−16^
	TRPV2	DNM1L	0.68	<2.2 × 10^−16^
		FIS1	0.99	<2.2 × 10^−16^
		MFF	0.88	<2.2 × 10^−16^
		MFN1	0.85	<2.2 × 10^−16^
		MFN2	1	<2.2 × 10^−16^
		OPA1	0.97	<2.2 × 10^−16^
	TRPM1	DNM1L	0.61	<2.2 × 10^−16^
		FIS1	0.35	<2.2 × 10^−16^
		MFF	0.47	<2.2 × 10^−16^
		MFN1	0.7	<2.2 × 10^−16^
		MFN2	0.63	<2.2 × 10^−16^
		OPA1	0.67	<2.2 × 10^−16^
	TRPM7	DNM1L	0.98	<2.2 × 10^−16^
		FIS1	0.71	<2.2 × 10^−16^
		MFF	0.87	<2.2 × 10^−16^
		MFN1	0.93	<2.2 × 10^−16^
		MFN2	0.98	<2.2 × 10^−16^
		OPA1	0.99	<2.2 × 10^−16^

These single-cell analysis revealed the comprehensive role of SOCs together with MDGs in the tumor microenvironment.

Overall, our *in silico* approach depicted that SOCs might be involved in the regulation of mitochondrial function in HNSC and the expression of SOCs along with the MDGs can be a predictive marker of HNSC and might have prognostic value in these patients.

## Discussion

Dysregulated intracellular Ca^2+^ signaling in cancer cells is shown to be remarkably associated with cancer cell growth, proliferation, angiogenesis, and metastasis (Barr et al., 2008; Chen et al., 2011; Wang et al., 2012; Bergmeier et al., 2013; Motiani et al., 2013; Wang et al., 2022). Dysregulation of SOCE and Ca^2+^ imbalance was reported in Sjögren’s syndrome and in head and neck cancers treated with radiation (Cheng et al., 2012; Ambudkar, 2018). Elevated serum calcium levels is a proposed diagnostic marker for head and neck malignancy (Bradley and Hoskin, 2006). Recently, Newton et al. (2020) demonstrated that monoclonal antibody against PD-L1 enhances the functionality of T cells by modulating calcium release-activated calcium channels (Newton et al., 2020). siRNA-mediated knockdown of ORAI1 and STIM1 in Ca9-22 and OECM-1 oral cancer cell lines showed reduced proliferation, migration, and invasion of these cells (Wang et al., 2022). In the current study, we revealed that SOCs might be involved in regular mitochondrial function, and alteration in these might be a predictive and prognostic marker.

Substantial evidence has been provided in several different types of cells that SOCs are involved in cell interaction and secretory Ca-ATPase-2 pathway (Bergmeier et al., 2013). In addition, recent evidence suggests that the formation of a complex of these proteins with phosphatase calcineurin dephosphorylates cytoplasmic NFAT and induces nuclear translocation. Nuclear NFAT transcriptionally activates the expression of several genes including NANOG, OCT4, SOX2, and FGF19 which are involved in cancer cell stemness (Wang et al., 2021). In the current study, we conducted the gene ontology analysis, as a preliminary analysis to show the involvement of SOCs in the regulation of IP3 and ATPase pathways. Due to the involvement of SOCs in signaling pathways apart from their regular transport activities we hypothesized that SOCs might be involved even in the regulation of mitochondrial activities. Furthermore, disease ontology analysis was conducted to show the involvement of SOCs in several cancers.

Earlier studies have shown that the entry of Ca^2+^ ions through store-operated channels begins with the stimulation of plasma membrane receptors to phospholipase C and synthesis of inositol triphosphate. Activated SOCE aid in refilling Ca^2+^ stores for further stimulation (Putney, 1986; Parekh and Putney, 2005). In the current study, disease ontology analysis showed their involvement in inflammatory diseases, eye tumors, and medulloblastomas. This is in accordance with earlier *in vitro* studies (Wang et al., 2022). Subsequently, the TCGA-HNSC mRNA expression of SOCs showed that STIMs, ORAIs, TRPCs, TRPVs, and TRPMs are significantly altered in HNSC patients. The survival analysis clearly demonstrated that alteration in SOCs mRNA expression remarkably decreases the survival rate of these patients. Further, TCGA-HNSC based correlation analysis using Timer 2.0 tool showed a significant correlation in the expression of MDGs with SOCs. Mitochondrial regulation of SOCE is due to the ability to rapidly uptake the Ca^2+^ thus modulating the inositol phosphate mediated signaling. Hoth et al. (1997) showed that mitochondrial uncouplers inhibited Ca^2+^ exit from mitochondria leading to the prevention of sustained entry of Ca^2+^ into T-cells (Hoth et al., 1997). Further, Gilabert and Parekh revealed respiring mitochondria are required for Ca^2+^ homeostasis by CRAC channels (Gilabert and Parekh, 2000). In addition, FDA-approved drugs, leflunomide and teriflunomide were shown to be inhibitors of SOCs at clinically-relevant doses in neuroblastoma cells (Rahman and Rahman, 2017). Miret-Casals et al. (2018) demonstrated that leflunomide and teriflunomide induces mitochondrial fusion through the activation of MFN2 in cervical cancer cell lines (Miret-Casals et al., 2018). Leflunomide has also been shown to promote mitochondrial fusion via downregulating total and phospho DNM1L and inducing MFN2 leading to growth retardation in pancreatic adenocarcinoma cells (Yu et al., 2019). Recently, Yedida et al. (2019) demonstrated that apogossypol (a small molecule inhibitor of pan-Bcl2) mediated endoplasmic reticulum (ER) reorganization results in Ca^2+^ transfer between ER and mitochondria leading to inhibition of mitochondrial fission and apoptosis of HeLa cells (Yedida et al., 2019). This study also showed that leflunomide, a potent inhibitor of SOCs, inhibits apogossypol-mediated ER reorganization and antagonizes its protective effect against apoptosis (Yedida et al., 2019). In accordance with these studies, our docking results showed higher negative binding energy for SOCs with MDGs. To our knowledge, there are no *in vitro* or *in vivo* studies showing the direct or indirect binding of SOCs with mitochondrial proteins. The STRING database also showed no known direct relation among these proteins. In the current study, we analyzed phosphoproteomic data for head and neck cancer from CPTAC. Mertins et al., 2016 integrated proteomics and phosphoproteomics data of CPTAC to identify distinct profiles in 77 genomically annotated breast tumors. In this study, the authors also revealed changes in phosphoproteomics of CDK12, PAK1, RIPK2, and TLK2. This study also proposed the phosphoproteomic changes in these proteins can be utilized as druggable kinases beyond HER2 (Mertins et al., 2016). Hence, we conducted the protein and phosphoprotein analysis for clinical samples to show SOC proteins are also valuable biomarkers alongside the mRNA expression in head and neck cancers.

Among the post-translational modifications, phosphorylation of the protein is central to signaling mechanisms and is critical for various physiological responses. There are about 50,000 known phosphorylation sites that do not currently have any ascribed functions (Mayya and Han, 2009). However, quantitative phosphoproteomics has been an effective tool to identify functional phosphorylation sites and putative substrates of kinases. Immunoprecipitation followed by phosphoproteomics using mass spectrometry demonstrated that RTKs including ALK, ROS fusion proteins, PDGFRα, and DDR were found to be highly phosphorylated in non-small cell lung carcinoma cell lines and tumor samples (Rikova et al., 2007). Recently, phosphotyrosine directed mass spectrometry analysis conducted by Van Linde et al. (2022) showed the complex kinase activities in glioblastoma. The study suggested the potential of phosphoproteomic analysis for the identification of targets for the treatment modalities (Van Linde et al., 2022). In the current study, analysis of the protein and phosphoprotein expression of SOCs were crucial to show that they might be involved in the regulation of mitochondrial dynamic changes. Also, high levels of phosphoprotein expression of SOCs indicate their activity in head and neck cancers. Further, the CPTAC-HNSC dataset revealed a significant correlation in the expression of SOCs with MDGs. The survival correlation analysis indicated that the patients expressing altered levels of SOCs along with MDGs possess less survival probability compared with those who are expressing normal levels of either SOCs or MDGs. This indicated that the evaluation of SOCs combined with MDGs might be a potential biomarker in HNSCs.

Furthermore, single-cell analysis showed heterogeneity in the expression of SOCs across the ten different cell types. The co-culture of mouse embryonic fibroblasts with MDA-MB-231 by Yang and Huang (2005) showed the critical role of Ca^2+^ ions influx in fibroblast cells migration (Yang and Huang, 2005). In addition, Davis et al. (2012) demonstrated that altered function of ORAI1 and TRPC1 led to EGF-induced EMT changes in triple-negative breast cancer cell lines (Davis et al., 2012). More recently, Zhang et al. (2020) revealed serum- and glucocorticoid-inducible kinase 1 (SGK1) regulates osteoclastogenesis via controlling ORAI1 leading to bone metastasis of breast cancer both in *in vitro* and *in vivo* models (Zhang et al., 2020). In accordance with these results, single-cell analysis described that the expression of SOCs were found to be strongly correlated with the expression of MDGs in cancer cells and fibroblasts indicating the role of SOCs in conjunction with mitochondrial dysfunction in driving the cancer cell hallmarks. Additionally, our analysis showed the critical role of tumor heterogeneity in the progression of cancer and highlights the importance of developing targeted therapy for the microenvironment niche. However, further studies need to be conducted to validate the role of differential expression of SOC in the different cell populations of the tumor tissue.

Taken together, these results indicated that SOCs and MDGs combined alteration might be a potential diagnostic and prognostic marker in HNSC.

## Conclusion

Our *in silico* analysis shows the altered mRNA and protein expression of SOCs in head and neck cancer and suggests their role as possible biomarkers. We also showed a strong correlation in the expression of MDGs with SOCs in TCGA-HNSC, CPTAC-HNSC, GSE171898, and GSE103322 datasets. We showed for the first time that the SOCs binds to MDGs with very high efficiency. Mechanistic studies need to be conducted further to validate their role in mitochondrial dysfunction and in HNSC development. Based on our *in silico* studies we propose that the expression of SOCs along with MDGs might serve as a better early diagnostic and prognostic marker in HNSC patients. However, further studies need to be conducted to evaluate their potential use in clinical diagnosis and management.

## Data Availability

Publicly available datasets were analyzed in this study. This data can be found here: NCBI Gene Expression Omnibus.

## Supplementary Material

Supplementary Figure 1 Graphical Abstract.
