# Supplementary figures and images for: Differential Expression of Genes Regulating Store-operated Calcium Entry in Conjunction With Mitochondrial Dynamics as Potential Biomarkers for Cancer: A Single-Cell RNA Analysis
Source: Front Genet. 2022 May 31;13:866473. doi: 10.3389/fgene.2022.866473 (PMC9197647; doi:10.3389/fgene.2022.866473)

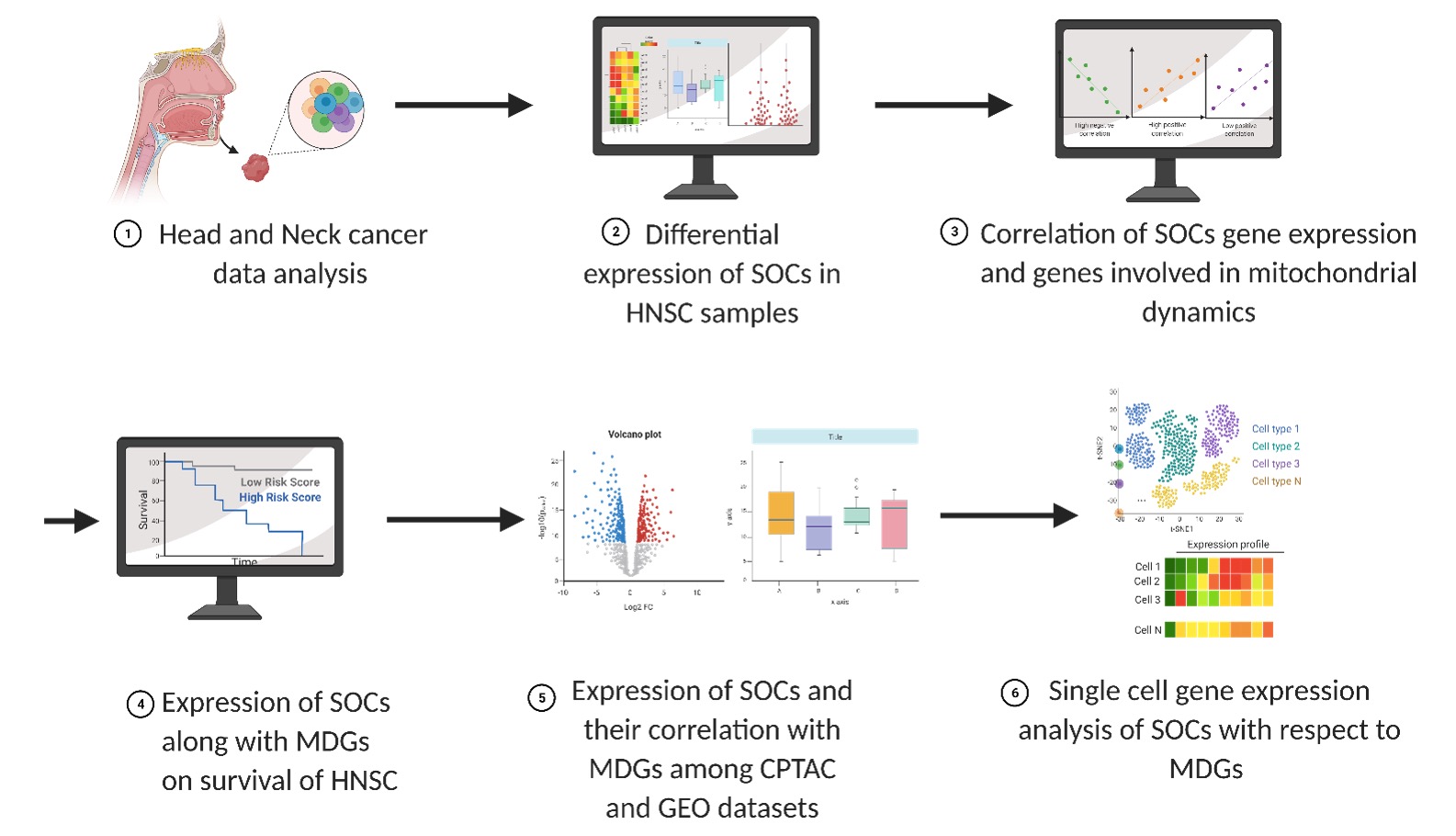

Supplement: Supplementary file 1 [file Image1.JPEG]
